# Understanding adaptations in the Veteran Health Administration’s Transitions Nurse Program: refining methodology and pragmatic implications for scale-up

**DOI:** 10.1186/s13012-021-01126-y

**Published:** 2021-07-13

**Authors:** Michaela S. McCarthy, Lexus L. Ujano-De Motta, Mary A. Nunnery, Heather Gilmartin, Lynette Kelley, Ashlea Wills, Chelsea Leonard, Christine D. Jones, Borsika A. Rabin

**Affiliations:** 1 Denver-Seattle Center of Innovation for Veteran-Centered and Value-Driven Care (COIN), VA Eastern Colorado Health Care System, 1700 N. Wheeling Street, P1-151 Research, Denver, CO 80045 USA; 2 College of Nursing, University of Colorado Anschutz Medical Campus, Aurora, CO USA; 3 Health Systems, Management, and Policy, Colorado School of Public Health, Anschutz Medical Campus, Aurora, CO USA; 4 Division of Hospital Medicine, Department of Medicine, University of Colorado Anschutz Medical Campus, Aurora, CO USA; 5 Herbert Wertheim School of Public Health and Human Longevity Science, University of California San Diego, La Jolla, CA USA; 6 UC San Diego Dissemination and Implementation Science Center, University of California San Diego, La Jolla, CA USA

**Keywords:** Adaptation, RE-AIM framework, Stirman adaptation framework, Implementation, Qualitative analysis, VHA

## Abstract

**Background:**

When complex health services interventions are implemented in real-world settings, adaptations are inevitable. Adaptations are changes made to an intervention, implementation strategy, or context prior to, during, and after implementation to improve uptake and fit. There is a growing interest in systematically documenting and understanding adaptations including what is changed, why, when, by whom, and with what impact. The rural Transitions Nurse Program (TNP) is a program in the Veterans Health Administration (VHA), designed to safely transition a rural veteran from a tertiary hospital back home. TNP has been implemented in multiple cohorts across 11 sites nationwide over 4 years. In this paper, we describe adaptations in five TNP sites from the first cohort of sites and implications for the scale-up of TNP and discuss lessons learned for the systematic documentation and analysis of adaptations.

**Methods:**

We used the Reach, Effectiveness, Adoption, Implementation, and Maintenance (RE-AIM) expanded version of the original Stirman framework to guide the rapid qualitative matrix analysis of adaptations. Adaptations were documented using multiple approaches: real-time database, semi-structured midpoint and exit interviews with implementors, and member checking with the implementation team. Interviews were recorded and transcribed. To combine multiple sources of adaptations, we used key domains from our framework and organized adaptations by time when the adaptation occurred (pre-, early, mid-, late implementation; sustainment) and categorized them as proactive or reactive.

**Results:**

Forty-one unique adaptations were reported during the study period. The most common type of adaptation was changes in target populations (patient enrollment criteria) followed by personnel changes (staff turnover). Most adaptations occurred during the mid-implementation time period and varied in number and type of adaptation. The reasons for this are discussed, and suggestions for future adaptation protocols are included.

**Conclusions:**

This study demonstrates the feasibility of systematically documenting adaptations using multiple methods across time points. Implementors were able to track adaptations in real time across the course of an intervention, which provided timely and actionable feedback to the implementation team overseeing the national roll-out of the program. Longitudinal semi-structured interviews can complement the real-time database and elicit reflective adaptations.

**Supplementary Information:**

The online version contains supplementary material available at 10.1186/s13012-021-01126-y.

Contributions to the literature

- The use of multiple methods of adaptation tracking and coding allowed for this analysis to be inclusive of multiple data points. This data triangulation provided a larger picture of program adaptations across time points.
- In response to limitations of a single framework, we tested an innovative method to document and analyze adaptations across various time points of the implementation continuum in a real-world setting through the use of the Reach, Effectiveness, Adoption, Implementation, and Maintenance (RE-AIM) framework and a modified version of the original Stirman framework.
- Research in health services has shown that adaptations rooted in local site context can influence sustainment. We realize it may be helpful to conceptualize and operationalize this earlier in the intervention period to support sustainment.

## Introduction

 Adaptations,  defined as modifications to an intervention, implementation strategy, or context, are inevitable when implementing complex health services interventions.  These can happen in a planned or proactive or an unplanned or reactive way before, during, and after implementation [1–3]. Previous work suggests that adaptations in complex interventions should be expected, embraced, and studied rather than suppressed and ignored [4]. Increasingly, more studies have focused on prioritizing, guiding, and studying adaptations over the past few years [3, 5–7]. Methodologies for the documentation and analysis of adaptations require further development, especially when it comes to understanding their impact on implementation and effectiveness outcomes.

Adaptations may vary depending on when they happen in the context of the implementation study. For example, adaptations occurring in the early stages of a project (pre-implementation or early implementation) may focus more on improving reach by changing rules around recruitment or adoption by adding or modifying strategies around engaging with sites and/or providers. Changes in the later stages of a project might be focused on preparing for or improving sustainment as ongoing management of the project transitions to the local implementation site [1, 8]. Finally, literature about the nature and impact of planned  (or proactive) and unplanned (or reactive) adaptations suggests a preference for planned adaptations but additional study is needed [2, 3, 6, 9, 10].

The Transitions Nurse Program (TNP) is a national quality improvement project designed to improve care coordination between urban tertiary Veterans Health Administration Medical Centers (VAMCs) and rural Patient Aligned Care Teams (PACTs). TNP has served over 4200 veterans nationally across 11 VAMCs [11] and increased visits to primary care providers (PCPs) at 14 days post-hospitalization and reduced the risk of death at 30 days post-discharge [12]. The implementation of TNP happened in two main cohorts. This paper focuses on adaptations from the five sites that comprised the first cohort.

The most prominently used framework that guides the documentation and analysis of adaptations was developed by Stirman-Wiltsey and colleagues in 2013 and was updated in 2019 [2, 3]. It provides a comprehensive coding system for adaptations and modifications. Our team used a modified version of the original Stirman framework as described by Hall et al. [8] which also includes considerations of impact as it relates to the constructs of the RE-AIM framework (i.e., Reach, Effectiveness, Adoption, Implementation, and Maintenance) [13, 14]. We previously piloted this modified framework to document adaptations across four Veterans Health Administration (VHA) health services programs [1]. The key constructs included in this framework are [15] elements that were changed, [1] the type of change, [2] the person(s) responsible for the change, [3] the timing of the change, [4] the basis for the change, [5] the reason for the change, (and 7) the short-term impact of the change. This modified framework provides a systematic, comprehensive method for capturing adaptations such as eligibility criteria and personnel changes. Structuring our matrix analysis around these constructs allowed us to examine the adaptations across both sites and time points.

The purpose of this paper is to describe the methods, analysis, and findings from our multi-method, multilevel adaptation documentation activity of the cohort 1 TNP implementation sites. Our objectives are [15] to describe the adaptations that occurred before, during, and after the implementation of the TNP project and their implications to future implementation and scale-up and [1] to discuss the lessons learned for the documentation and analysis of adaptations from a methodological perspective.

## Methods

We used a mixed methods approach to conduct a rapid qualitative matrix analysis to analyze the rich data that emerged.

### Intervention

TNP was designed around four core intervention components that remained constant across sites: [15] assess patient discharge readiness at the bedside, [1] engage with primary care providers and PACT clinics to make them aware of their patients’ admission, [2] coordinate a PCP follow-up appointment for the veteran within 14 days of discharge, and [3] complete the care coordination process by contacting the veteran within 72 h of discharge [11]. The program was informed by the Practical, Robust, Implementation and Sustainability Model (PRISM) [16] which guided the implementation while RE-AIM guided the evaluation of TNP [10]. Each TNP team consisted of one full-time nurse (or two part-time nurses) and one hospitalist site champion. The TNP nurse was situated at each VAMC site to engage the patient, the hospital care team, and the PACT to ensure a smooth and successful transition home for the veteran. Additionally, one hospitalist site champion was recruited per site to help engage physicians and hospital leadership. The TNP teams were encouraged to identify the inclusion/exclusion criteria specific to their site, provided that the patients met the criteria of hospitalization in an urban VAMC and a subsequent discharge to home. The four core components of the intervention were unchanged across sites. Key implementation strategies were employed across all sites including facilitation, centralized technical assistance, and ongoing consultation [12].

### Settings

TNP was developed and pilot tested at a single, large VAMC in the Rocky Mountain Region [17], and due to promising results, TNP was funded for expansion to additional sites in year 1 (cohort 1) and year 2 (cohort 2) [11, 15]. One cohort 1 site did not collect data on adaptations, so for this paper, we analyzed the adaptation data from the five cohort 1 sites that tracked adaptations and completed the program. TNP sites were located in five different states, and each site included an urban VAMC and multiple affiliated rural PACTs. Each site was required to enroll at least 50% rural veterans in the TNP intervention. VAMCs were eligible to enroll in TNP if they were located in an urban setting and coordinated patient care with rural PACTs [10]. Additional details about the TNP, its components, and specific sites have been published elsewhere [11, 18].

### Data collection and sources

We used three primary data sources to track adaptations within sites throughout the program: TNP database, semi-structured interviews (MK and MM), and member checks. TNP nurses used the TNP database to enter site adaptations (e.g., enrollment criteria) in real time. Adaptations to the components of the study that were not part of the four core components were allowed due to the pragmatic nature of this quality improvement project. This applied to the eligibility criteria as well. TNP nurses were instructed to document these adaptations in the TNP database. Primary eligibility criteria including discharge from the hospital to home and follow-up care with a VA primary care physician were required. Fidelity to the four core components of the intervention was maintained across sites [18]. The TNP database included open fields that corresponded to our theoretical framework [2, 3, 13, 14]. TNP nurses received a brief training on the use of the TNP database by the primary implementation team (LK and AM) who were available to provide ongoing technical assistance. All TNP nurses were encouraged to enter information into the TNP database on a weekly basis and received reminders during regular team meetings. Our implementation team sent reminders to the TNP nurses about the use of the TNP database on a monthly basis. The template for the TNP database is provided in the supplemental materials (Additional file 1: Appendix 1).

Purposive semi-structured interviews were conducted during implementation about one year into the implementation of the TNP program (mid-line interview), and another interview was conducted at the end of the program (exit interview), approximately two years after the implementation. These interviews were conducted over the telephone with TNP nurses and site champions (i.e., implementors) and were specifically designed to document adaptations using our modified Stirman/RE-AIM theoretical framework. All nurses and site champions were eligible for the interviews. It is unknown whether data saturation was reached as the site implementors were the only interviewees. Transcripts were not returned to the interviewees for comment. Interview guides for the mid-line interviews were adapted to the TNP project from the generic guide developed and described in Rabin et al. [1] Full interview guides specific to the TNP project are provided in the supplemental materials (Additional file 2: Appendix 2). The same eight adaptation questions from the mid-line interview were also asked at the end of the exit interview. These questions were prefaced with inquiries about their experience in their role (i.e., transitions nurse or site champion), plans for sustainment of the program, and changes in their site’s process map. Interviews were audio-recorded and transcribed verbatim. The mid-line and exit interviews lasted on average 30–40 min. The third data source was member checks with key members of our training and implementation team to verify if any adaptations were missing and to validate data coding. These occurred at team meetings during the analysis phase and their responses were documented in meeting minutes.

### Data management and analysis

**Fig. 1:**
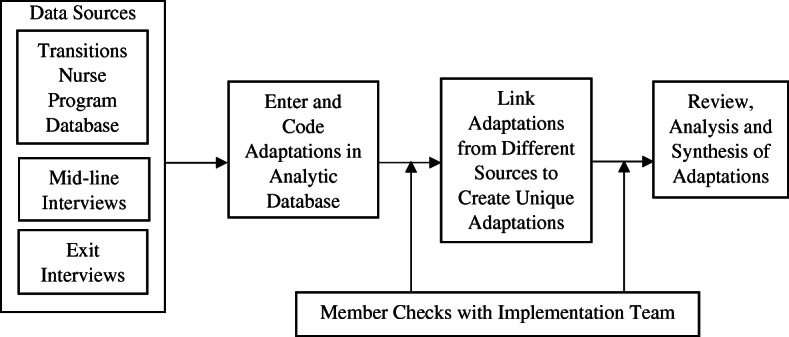
Process of identifying and analyzing adaptations in the Transitions Nurse Program

To organize our data from various sources, we created a centralized data abstraction database (analytical database) guided by our modified Stirman/RE-AIM theoretical framework [2, 3, 13, 14]. We further expanded the analytic database with additional data fields that identify details of the adaptation (i.e., site, interview subject), source (TNP database, mid-line interview, exit interview, member check), and timing (i.e., pre-implementation; early, mid-, and late implementation; sustainment), following the structure of the Quality Enhancement Research Initiative (QUERI) Roadmap [19]. Figure 1 (process of identifying and analyzing adaptations in the Transitions Nurse Program) shows the data sources and analytic processes. Pre-implementation was defined as project setup prior to patient recruitment, early implementation was defined as the first 3 months, mid-implementation was defined as months 4–23, late implementation was defined as months 24–30, and sustainment was defined as changes occurring after the implementation of TNP was completed.

Information from the TNP database and the coded interviews were compiled in our analytic database. The integration of this data was relatively straightforward as the TNP database, the interviews, and the abstraction within the analytical database were all structured based on our modified RE-AIM/Stirman framework. The TNP database was cleaned, and additional identification fields were completed by the primary analyst (LUD) and checked by a second (MN). Validated TNP database entries were compared to transcripts of ongoing monthly calls between the implementation team and the TNP nurses to triangulate and member check each TNP database entry.

Mid-line and exit interviews were transcribed and cross-checked with the audio files. Interview transcripts were inductively coded using a rapid matrix analytic approach where pre-defined categories from the data were directly abstracted into the analytic database with supporting quotes [20]. Each adaptation identified in an interview transcript was entered into the analytic database by a qualitative analyst (MM) along with its source and related information about the adaptation as defined by the modified adaptation framework (e.g., elements, type of change, timing, impact). Each initial entry of an adaptation was validated by an additional analyst (MN). Two members of the implementation team (LK and AM) then verified the entries to ensure completeness of the data. When questions emerged around categories, we used the expertise of the implementation team to member check and validate the decisions.

Once all adaptations were entered into the analytic database from both the TNP database and the interviews, entries that referenced the same adaptations in different sources, time points, or by different stakeholders were linked so unique adaptations could be identified. For this process, two analysts (MM and MN) identified linked adaptations independently and the research and implementation team resolved differences and validated decisions as needed.

Types of adaptations were identified using the established sub-categories: elements, what was changed, who was responsible for the change, how or on what basis was the change made, why the change was made, and the short-term impact of the adaptation. All adaptations were coded as proactive and reactive by two analysts independently (MM and MN). For the purpose of this analysis, proactive adaptations were defined as strategized by the intervention team, and reactive adaptations were defined as originating outside the intervention team, responding to a change in the setting or broader context that required modifications to the intervention delivery and implementation. Differences in the initial coding for this category were resolved by the senior author (BR). A further post hoc analysis was conducted to better understand the adaptations that were coded as “other” in the “What was changed” construct category. Two analysts (MM and MN) used a grounded theory approach to review these adaptations and to deductively determine emerging codes and identify themes. This paper is guided by the Consolidated Criteria for Reporting Qualitative Research (COREQ) reporting standards [17].

## Results

Adaptations were tracked using the TNP database and mid-line and exit interviews with TNP nurses and site champions. Fourteen total entries in the TNP database occurred across four of the five sites. Eight mid-line interviews and 10 exit interviews provided additional TNP adaptation information. The number of interviews conducted varied across sites at mid-line with most sites having conducted two interviews except for sites 1 and 3 where interviews were only conducted with the TNP nurse and not the site champion. A total of 49 identified adaptations were entered into the analytic database from all sources, and after merging duplicate adaptation categories, 41 unique adaptations were identified. The number of adaptations identified from all sources ranged from six (site 2) to 13 (site 5) across the sites, with six (site 2) to 10 (site 5) unique adaptations identified. Site 2 had the fewest adaptations identified and with no adaptations entered into the TNP database. Most adaptations were identified through the exit interviews [13], followed by the TNP database [13] and the mid-line interviews [9]. One adaptation (i.e., hospital move) was not identified through any of the sources but was captured during member checking with the implementation team. No adaptation was mentioned across all three sources. All results in the tables are reported as the number of unique adaptations (see supplemental material [Additional file 3: Appendix 3] for the detailed distributions of adaptations across data sources).

The distribution of the unique adaptations across sites and time points (i.e., pre-implementation, early, mid-, and late implementation and sustainment) is described in Table 1. Most adaptations were identified as occurring during mid-implementation. Six adaptations were in early implementation, thirty in mid-implementation, and four in late implementation. Only one adaptation (personnel change) occurred pre-implementation and none during sustainment. No substantial variations of across sites for these distributions was observed across sites.

**Table 1:** Distribution of unique adaptations across sites and time points

Timing of adaptations across sites and time points					
Phase	Pre-I	Early-I	Imp	Late-I	Sustainment
Site 1	0	0	5	3	0
Site 2	0	2	4	0	0
Site 3	0	0	7	1	0
Site 4	0	1	8	0	0
Site 5	1	3	6	0	0
**Total**	**1**	**6**	**30**	**4**	**0**

*Pre-I* pre-implementation, *Early-I* early implementation, *Imp* implementation, *Late-I* late implementation, *Sustainment*

### Type of adaptations

Adaptations were characterized by the constructs and sub-categories of our modified Stirman/RE-AIM adaptation framework. Adaptation constructs that will be discussed here include primary element that was changed, what was changed, who was responsible for the change, how or on what basis was the change made, and why the change was made (see Table 2).

**Table 2:** Unique adaptations identified across time points and various constructs of the modified adaptation framework

Adaptation constructs	Pre-I	Early-I	Mid-I	Late-I	Sustainment	Total
**Elements**						
Format	0	0	2	1	0	3
Personnel involved	1	0	7	1	0	9
Target population	0	4	16	2	0	22
Intervention presentation	0	2	4	0	0	6
Others	0	0	1	0	0	1
**What was changed**						
Tailoring to individuals	0	0	3	2	0	5
Adding a component	0	0	0	0	0	0
Removing a component	0	0	0	0	0	0
Condensing a component	0	0	0	0	0	0
Extending a component	0	0	1	0	0	1
Substituting for a component	0	0	1	0	0	1
Changing the order of components	0	0	0	0	0	0
Integrating with other programs	0	3	1	0	0	4
Repeating a component	0	0	0	0	0	0
Loosening the structure or protocol	0	0	0	0	0	0
Otherwise changing the intervention	1	3	24	2	0	30
**Who was responsible for this change**						
Entire or most of the team	0	3	9	0	0	12
Provider (TN/SC)	1	3	16	0	0	20
Administrator	0	0	3	1	0	4
Researcher	0	0	0	3	0	3
Developer	0	0	0	0	0	0
Stakeholder	0	0	1	0	0	1
Coalition	0	0	1	0	0	1
**How or on what basis were these changes made**						
Based on our vision or values	0	0	1	2	0	3
Based on a framework	0	0	0	0	0	0
Based on our knowledge or experience	0	1	7	0	0	8
Based on QI data, summary information, or results	0	0	0	0	0	0
Based on pragmatic/practical considerations	1	3	18	1	0	23
Based on financial incentives/payments	0	0	0	0	0	0
Based on feedback or suggestions	0	1	3	1	0	5
Others	0	1	1	0	0	2
**Why was the change made**						
To increase the number or type of patients contacted (Reach)	0	1	10	3	0	14
To enhance the impact or success of the intervention for all or important subgroups (Effectiveness)	0	2	6	0	0	8
To make it possible to involve more teams, team members, or staff (Adoption)	0	0	0	0	0	0
To make the intervention delivered more consistently; to better fit our practice, patient flow, or EHR; for practical reasons (Implementation)	0	2	7	1	0	10
To save money or other resources (Implementation)	0	0	0	0	0	0
To institutionalize or sustain the intervention (Maintenance)	0	0	0	0	0	0
To respond to external pressures or policy	0	0	1	0	0	1
Others	1	1	6	0	0	8
**Short-term impact of the change**						
Positive	0	5	18	3	0	26
Negative	0	0	3	0	0	3
Unknown	1	1	9	1	0	12

*Pre-I* pre-implementation, *Early-I* early implementation, *Imp* implementation, *Late-I* late implementation, Sustainment

#### Elements

The two most common element adaptations were recruitment (target population) and personnel (personnel involved). Recruitment adaptations (n = 22, 54%) primarily captured changes to the enrollment criteria by site, but also included prospective patient identification and outreach to different outpatient providers. Enrollment criteria were most often expanded, but in some cases, it was restricted to exclude populations already receiving similar care within existing site practices. The following are examples for expanding:

…I was finding that I was doing just as much work for these [non-TNP] patients… so it was like why not, why not put these people in the program, too? – TNP nurse

Yes, I know we’ve kind of tinkered with our admission criteria. We had you know certain, I think where we really struggled as a site was well, what’s considered rural and what’s not, and once we kind of worked on clarifying that, that basically it was anyone that wasn’t seen at our main hospital for primary care could be included in our program, that was actually a lot easier. – TNP nurse

The following are examples for restricting:

For example, we exclude oncology patients because the oncology service already has two nurses that function in a similar role. – site champion

Personnel changes were the second most commonly reported element (n = 9, 22%). These included changes at all personnel levels, including TNP nurses, site champions, and executive leadership, and often reflected the challenges that personnel turnover presents to implementing and sustaining an intervention. The only pre-implementation adaptation documented in this analysis was a site champion personnel change that occurred before the program began enrolling patients. Most documented personnel changes were in the mid-implementation phase, although one late-implementation change was reported.

We started out- our first TNP nurse was somebody who was perfect for the role and extremely motivated… [then with the second TNP nurse] it took a little while to properly implement the intervention. – site champion

#### What was changed/type of change

The second area of TNP adaptations explored the primary type of change involved as part of the adaptation. The qualitative coding indicated that the most adaptations were coded as otherwise changing the intervention (n = 25, 61%). The two main themes that emerged from this analysis were also changes to recruitment (n = 19; 63%) and personnel (n = 9, 30%). Subtle differences emerged during the post hoc analysis and subthemes. For example, recruitment changes included forming relationships, rounding with inpatient hospital teams, and increasing outreach to primary care providers.

I’m really working closely with case management, inpatient case management here… It just seemed to happen over time that it seemed like a better fit, identifying high risk individuals that would benefit from the TNP program. – TNP nurse

A subtheme of personnel changes was the repeated acknowledgement that personnel turnover across all levels (TNP nurse, site champion, leadership) resulted in a decrease in immediate productivity and a likely negative effect on sustainment.

And in our case, I was chief of medicine at the time this started so as the leader of the medicine service line I was clearly engaged and in support of it, but in the end I’m no longer in that role, the nursing supervisor that was in his role is gone, the director was not really engaged even though she said she was. – site champion

#### Who was responsible for the change

Two groups emerged as responsible for most of the changes that occurred: local clinicians (n = 20, 49%), defined as site-specific TNP nurse and/or site champion, and the broader TNP team (n = 12, 29%), including the local TNP nurse, site champion, and the primary implementation team (LK and AM). Often the clinicians described using site-specific contextual knowledge as the reason to adapt the intervention. Most of these changes were made during the mid-implementation phase (n = 31, 76%) as TNP nurses and site champions refined aspects of patient recruitment and established working partnerships with hospital-based teams and community-based primary care providers.

Our pharmacist does med recs (reconciliations) on every single patient that’s discharged, so that’s a nice benefit for nurses because we don’t have to do that, and even in my role, in the TNP role, I don’t necessarily have to do med recs. I do go over meds… but primarily meds that may be new. – TNP nurse

Three adaptations were suggested by the TNP implementation team in response to systemic changes at one site. Four adaptations originated with local hospital administration and included changes to personnel and the target population. The adaptations decided on by the entire or most of the team often originated with a clinician consultation with the implementation team, either individually or through monthly team meetings.

We had noticed that our criteria was a little narrow and enrollment was slow. A member of [the implementation team] suggested we reexamine our criteria. – TNP nurse

#### How or on what basis was the change made

Pragmatic or practical considerations (n = 23, 56%) were the reason most adaptations were made, although TNP nurse knowledge or experience of working with patients (n = 8, 20%) was also prevalent. Most changes were made during mid-implementation (n = 31, 76%) which mirrors and may be related to who was making the change.

I know we have the template that was given to us, but …every facility is different, like for me… I had to create a different handoff communication to send to my PACT nurses, but you know, every facility is different. – TNP nurse

I think they opened [the enrollment criteria] up and said that the specialty cares could do the follow up visit. And so, I’ve done that sometimes. You know, where a patient is in for [medical condition] and he’s not going to go to the clinic that’s fifty miles away from his home, when he’s got to come back in to [site] for specialty care follow up. And so, we used that as a follow up. – TNP nurse

The adaptation identified only by member checking with the implementation management team was based on pragmatic concerns. At one TNP site, the hospital moved locations during the course of the study. Necessarily, the census was lowered to accommodate this move, and TNP recruitment was affected.

#### Why the change was made

The most commonly reported reason was “to increase the number or type of patients contacted” (n = 14, 34%), followed by “to make the intervention delivered more consistently; to better fit our practice, patient flow, or EHR; and for practical reasons” (n = 10, 24%). An example of this reasoning is presented below.

Patients were screened to see if they were already enrolled in a discharge program..[because].. it would’ve been duplicative to have those patients also enrolled with the Transitions Nurse Program. – site champion

Another example cited was the TNP nurse-initiated rounding with medical teams to better understand individual patient needs.

I think it, it does connect her with the teams a little bit more when she goes and I think she has found it useful, at least part of the time. – site champion

#### Short-term impact of the adaptation

We found that short-term impacts (positive/negative) of adaptations were not consistently documented across the adaptations. Few interviewees directly stated a short-term impact (n = 4, 10%). A couple of examples described situations where the adaptation positively impacted reach through increasing the number of patients enrolled in the program:

Well, I don’t know, her initial enrollment was lower, and then ever since then, I think in the last four months she’s been pretty consistent across the board, near 20 a month. – site champion

Another adaptation had a positive impact on TNP nurse satisfaction as it clarified recruitment goals and roles:

So, the short-term impact, if it leads to more job satisfaction for the transitions nurse because it better defined what her goals were. … Who her target population was. It eliminated a lot of ambiguity because with our wealth of patients, it could’ve otherwise been overwhelming. – site champion

In other interviews (n = 9; 22%) the short-term impact was not specifically addressed but could be inferred. For example, in the interview below, the analysts coded this as a positive short-term impact, based on the respondent’s words.

It gave one nurse just more autonomy, and it kept things just like the flow was better, as far as what had taken place the day before, who still needed to be called, and I think it just probably helps with patient care. – site champion

Short-term impacts of other adaptations (n = 21, 51%) were estimated and coded by analysts (MM and MN), although several did not provide enough information to be assigned a code (n = 7; 17%). Overall, most adaptations were positive (n = 26, 63%), and three personnel changes were identified as negative (7%). Adaptations were additionally coded as proactive (n = 30, 73%) or reactive (n = 11, 27%). Proactive adaptations were largely related to enrollment criteria (n = 23, 56%) as TNP teams worked to tailor the intervention to best fit the population and contextual needs of each site. Personnel changes/staff turnover was the most common reactive adaptation (n = 8, 20%) and included TNP nurse, site champion, and leadership changes.

## Discussion

We used a RE-AIM-enriched version of the original Stirman-Wiltsey adaptation and modification framework [2, 3] and the QUERI Roadmap [19] to guide the systematic documentation, analysis, and interpretation of adaptations across a multi-site national care coordination program. Longitudinal and multi-stakeholder database entries and interviews were used to assess adaptations across five sites over 3 years. This provided both real-time and reflective understanding of site-specific adaptations and allowed us to compare within and between sites.

Collecting data at different time points from different stakeholders and different sources allowed us to triangulate the data for a richer understanding. Additionally, we found that member checking with the main implementation team provided rich contextual details that were not reflected in the database and interviews. For example, at one site, enrollment criteria were changed to allow patients with a non-VA primary care provider to enroll in TNP. The change was reported by the TNP nurse in a mid-line interview, and the main implementation team was able to provide context that the change was in response to the VA MISSION Act [18] which allows veterans to seek care in the community. Similar to our member checking approach, Finley and colleagues also found that the use of periodic reflections by the implementation team during the implementation of evidence-based programs in the VHA and beyond was helpful [19].

An important related finding from our analysis was that no adaptation was mentioned across all three sources (i.e., TNP database, mid-line interviews, and exit interviews) in this study. Furthermore, we identified one more adaptation not previously captured through member checking. These findings underline the importance of using multiple methodologies at different time points to collect information about adaptations and to triangulate these data sources to obtain a full picture of adaptations in real-world studies [21]. An important challenge is to strike the right balance between getting an accurate, full picture of adaptation in the study and the burden of data collection placed on the clinical research team and implementers.

We observed a change in the type and the intention of TNP adaptations depending on when these adaptations happened. More specifically, we noted the adaptations happening in the early stages of the project (early or mid-implementation) focused on improving reach by changing rules around recruitment or adoption by adding or modifying strategies around engaging with sites and/or providers. This is consistent with earlier TNP findings in which changes to enrollment criteria emerged as a common adaptation in early implementation [11]. In the later stages of the project, TNP adaptations were focused on preparing for or improving sustainment by editing protocols to customize the intervention to the local VAMC. In the future, it may be helpful to address possible protocol edits earlier in the intervention period to support sustainment.

Personnel turnover was a common TNP adaptation. Turnover occurred across roles (TNP nurse, site champion, leadership) and over different time points from pre-implementation to late implementation. This required the remaining team members to be resilient. Most TNP nurse changes were discussed as negative, requiring additional training, temporarily decreasing patient recruitment, and impacting program implementation. Site champion and leadership changes were reported to affect institutional buy-in and sustainment more than implementation. A prior study by Woltmann and colleagues emphasized the importance of using compensatory strategies to counterbalance the impact of staff turnover on the implementation of evidence-based programs in clinical settings [20]. Future studies should consider compensatory strategies across implementation stages to support site buy-in beyond individual personnel.

We found that adaptations are heavily influenced by personnel and context, often in interplay with each other. Few adaptations that were identified occurred in isolation. For example, experienced and resourceful TNP nurses across sites often started rounding with hospital teams during implementation, which may have changed recruitment criteria and influenced the type of patients that were enrolled in TNP. In turn, this local adaptation may have strengthened teamwork, increasing overall patient enrollment as the TNP nurse embedded within the hospital team.

In our analysis, 73% of adaptations were coded as planned (proactive) and 27% as unplanned (reactive). While the general guidance in the field is to avoid reactive, unplanned adaptations [6, 9, 10], in our study, we found that many of these changes were often hard to anticipate, contextually driven, and out of the control of the research or implementation team. However, we also found that often these unplanned changes did not lead to negative implementation outcomes as the implementation team and clinic partners were able to thoughtfully assess the changing context, practice great resilience, and creatively adjust the delivery strategies to match the new situation. Our findings suggest that it would be important to revisit our assumption whether unplanned or reactive adaptations are more likely to lead to less favorable outcomes than proactive changes.

Systematically documenting the impact (positive or negative) of adaptations on process and effectiveness outcomes as well as sustainment proved challenging. In this study, we attempted to collect information about the impact of adaptations using a qualitative approach asking about the short-term impact of the adaptations as it relates to various RE-AIM outcomes of reach, effectiveness, adoption, implementation, and maintenance. When asked about the impact of adaptations, TNP nurses and site champions often did not feel comfortable gauging whether the change might have resulted in improved or worsened outcomes. Furthermore, at the time of the interviews, interviewees did not have access to objective, quantitative data to answer this question. Evaluating the impact of adaptations is an area of great need for the field, and most adaptation frameworks do not provide guidance on how best to capture the impact of adaptations on diverse outcomes [10]. This could be assessed in near real-time through involving clinical staff with the implementation team. To address the gap in proper guidance on evaluating the impact of adaptations, Kirk and colleagues proposed a new adaptation model, the model for adaptation design and impact (MADI) [10]. In a recent study, Aschbrenner and colleagues evaluated the impact of agency-led adaptations on a lifestyle program for adults with serious mental illness and found that adaptations, when maintaining the core function of an intervention, can positively impact patient outcomes [21]. An innovative approach to identify what combination of adaptations lead to positive change in colorectal screening outcomes was proposed by Coury and colleagues [22]. Their study applied a qualitative comparative analysis approach and concluded that various combinations of adaptations led to positive outcomes with the size of the health system emerging as an important contextual factor.

A critical next step for the field is to systematically plan for and collect data on the impact of adaptations across studies.

We found some methodological challenges in our documentation and analysis of adaptations. Some extraction categories were challenging to define and implement in interviews and analysis. This required extensive discussion among team members to come to a consensus definition of categories, which may not be representative of how other analyses use similar categories. For example, we had an extensive discussion to interpret the meaning of the various sub-categories of the “how or on what basis were these changes made” and how to deal with situations where multiple sub-categories might apply, emphasizing that frameworks need to be adapted and carefully operationalized for each study context.

This project had limited success in capturing pre-implementation and sustainment adaptations. Each site was encouraged to adapt enrollment criteria pre-implementation to best fit their patient population and local context. Adaptations were discussed between sites and the implementation team during early support/mentoring calls and subsequent monthly group calls. Minutes were taken during these calls, but extracting adaptations-specific data from this secondary source was determined as less reliable than the TNP database and interview data, and therefore was not utilized for this analysis. Due to the timing of the exit interview, adaptations that occurred following this time point were not systematically collected and not included in this analysis. To acquire this data, future projects could incorporate a planned post-sustainment survey to assess adaptations further into the sustainment phase. Based on our team’s experience with this analysis, we are developing and implementing anadaptation tracking database in the early stage of future implementation projects to better capture pre-implementation adaptations.

Furthermore, while adaptations were tracked in near real time using the TNP database, they were not systematically used to inform further modifications to implementation. For future projects, we are developing and testing pragmatic ways to use information about adaptations real time to inform additional changes across implementation stages. For example, Glasgow and colleagues recently published a methodology (i.e., iterative RE-AIM process) that guides a more systematic data-driven approach to adaptations during implementation and encourages implementation teams to rely on process data from the RE-AIM framework (i.e., reach, adoption, implementation, effectiveness, and maintenance) to plan adaptations and monitor the incremental impact of these adaptations [13].

## Recommendations for researchers

A number of recommendations emerged from our work for researchers who are interested in systematically documenting and evaluating adaptations in their research projects:

1. There is an increasing appreciation of the importance of documenting, analyzing, and interpreting adaptations in research projects. Researchers should consider identifying and using pragmatic methods to systematically document adaptations in their research projects.
2. It is likely and expected that the type and number of adaptations will vary across different time points of a research project. To capture diverse adaptations, it is important to document them across the lifetime of a research project starting with pre-implementation through implementation and sustainment.
3. Adaptations are best captured using multi-method approaches and triangulation of data from different sources and from the perspective of multiple stakeholders. These methods can include interviews, surveys, observations, checklists, process maps, and other methods that best fit with the context of the research project. Member checking can further specify and enrich our understanding of adaptations.
4. The impact of adaptations in research projects has not been systematically and routinely assessed. Research projects should consider methods that can capture the impact of adaptations on implementation (e.g., reach, adoption, implementation, maintenance) and effectiveness outcomes.

## Conclusions

In summary, we found that the adapted Stirman/RE-AIM framework [1, 2, 13] provided a promising tool for the evaluation of adaptations across our program. We were able to systematically document and analyze adaptations across five sites over three years using both real-time and reflective data. Multiple sources of longitudinal data provided information about local contextual adaptations and common adaptations between sites. We were able to identify types of adaptations and the implementation phases in which they were made. We provided recommendations for researchers to consider for the documentation and analysis of adaptations. Future studies should expand on capturing the impact of adaptations and further test this model to establish its utility in practice.

## Supplementary Information

**Additional file 1.** Appendix 1. Real time TNP database structure.

**Additional file 2.** Appendix 2. Interview guide for midline interviews.

**Additional file 3.** Appendix 3. Overall adaptations and unique adaptations per site and per data source.

## Data Availability

The datasets generated and/or analyzed during the current study are not publicly available due to the Department of Veterans Affairs’ regulatory compliance.

## Abbreviations

MADI: Model for Adaptation Design and Impact
PACT: Patient Aligned Care Team
PCP: Primary care provider
QUERI: Quality Enhancement Research Initiative Roadmap
RE-AIM: Reach, Effectiveness, Adoption, Implementation, and Maintenance
TNP: Transitions Nurse Program
VA: Department of Veterans Affairs
VAMC: Department of Veterans Affairs Medical Center
VHA: Veterans Health Administration
