# Supplementary material for: Understanding adaptations in the Veteran Health Administration’s Transitions Nurse Program: refining methodology and pragmatic implications for scale-up
Source: Implement Sci. 2021 Jul 13;16:71. doi: 10.1186/s13012-021-01126-y (PMC8276503; doi:10.1186/s13012-021-01126-y)
Supplement: Supplementary file 2 — Additional file 2. Appendix 2. Interview guide for midline interviews. [file 13012_2021_1126_MOESM2_ESM.docx]

**ADAPTATIONS INTERVIEW QUESTIONS: TNP PROJECT / Midline**

**Overview:**

This is a structured interview with key staff implementing TNP program, to be conducted about 1/3 of the way through the program and shortly after the conclusion of the program. The key informants are to be identified with research staff on each project, but should include at least 3 staff who have different implementation roles (e.g. front lines implementer, supervisor, etc.). The interview is organized around user-friendly questions about the ‘who, what, when, how and why’ issues related to program delivery and adaptations that arise. The interview draws from various frameworks and adaptation models including Stirman et al (Stirman, 2013), RE-AIM (Gaglio et al, 2013; Glasgow et al, 1999), the ‘adaptome’ concept (Chambers and Norton, 2016), and prior interview experience.

**Introduction to Interviewees and Framing for Interview:**

My name is [interviewer name] and I am part of the TNP team in Denver, CO. One goal of this program is to explore the changes and modifications that have been made to the TNP program as it is being implemented at your site and across several VA medical centers. In the current phase of this project, we are interviewing site team members most closely involved with implementing the program at your site, such as yourself.

The findings of these interviews will inform the implementation of the program in the next round of sites. Additionally, we will be reporting these findings to the Office of Rural Health and share in scientific publications and gatherings. We anticipate this interview may take approximately 30-60 minutes.

We will not identify you individually in our report. Your responses will be kept confidential. Your participation in this interview is voluntary. You can stop the interview at any time, and let us know if you’d rather not answer a question. Do you have any questions before we begin?

In order to make sure we capture all of the information you give us, we would like to record this call. The audio-file for the recording will be uploaded to a restricted access file on the VA intranet immediately after we complete this interview. The recording will not include any identifying information, such as your name. Is this okay with you? If yes, I will ask you this question again once the audio recording begins.

We will now begin the audio recording: I have three questions for you.

1. Are you willing to participate in this interview?
2. Do you give us permission to record this interview?
3. And, do you give us permission to contact you at a later time if we have additional questions?

***[Generic prompts: If responses are limited or require clarification, probes may be used to illicit more detailed responses. Probes should use words or phrases presented by the participant using one of the following formats:***

*1. What do you mean by ____________?*

*2. Can you tell me more about ____________?*

*3. Can you give me an example of ____________?*

*4. Can you tell me about a time when ____________?*

We are interested in your experience conducting the TNP program. In particular, we are concerned about any **adaptations or modifications** that were made to the program over time, beginning with the initial planning up to the current time. We know that adaptations are common and conducted for various reasons and want to learn about them in your program.

By adaptation we mean *any changes to the TNP program intervention, protocol, or how it was delivered, whether intentional or unintentional.* Stated differently*, any changes made to your original plans*. One of the examples could be changing your enrollment criteria to meet the enrollment goals. Think back to how you originally understood the program at the training in Denver, and how, if at all, it might be most different now.

We would first like to have you list what you see as the major adaptations or modifications to the program made. Please list what you see as the most major or important adaptations first. After we understand the nature of the adaptation made, we will then ask a series of more detailed questions about the who, what, when, how and why of 2 or more of these adaptations, depending upon our time.

So, please list what you see as the major adaptations that have been made to the TNP program thus far:

**Note to the interviewer: probe to get basic idea of adaptation and select at least two that seem most significant. Consider the following examples of the TNP process to probe on:**

- **Eligibility**
- **Processes or timing of care – such as identifying patients, enrolling them, getting them ready for discharge, calling them after, interacting with PCP**
- **Working with the database**
- **Interacting with the champion/pact sites/hospitalists, etc.**
- **Staff responsible for various tasks**

Interview Questions

Now, I have several brief, follow-up questions about each adaptation. If you are not clear what I am asking, please let me know. Let’s begin with the first adaptation… (Repeat sequence of questions below for other adaptation(s).

1. WHAT Part 1: WHAT component or part of the intervention was changed in this adaptation; in other words, what was the nature of the change?

PROBE: For instance, was it a change to program content, format, delivery mode, staff delivering it, patients eligible, where, when or how it was delivered, or what? (**Record response in Table below and circle initial thoughts about which category in WHAT domain best fits**).

1. WHAT Part 2: How would you describe the *type* of change involved in this adaptation?

PROBE: Specifically, what did the change involve? Was something added, deleted, changed to better fit the patients, delivered at a different time or in a different way? (**Record response and circle initial thoughts about ‘which category in WHAT domain; the WHICH subcategory in Table below best fits).**

1. WHO was responsible for first suggesting or initiating this change?

PROBE: Was this the person or persons the ones who implemented the change? (If not, who implemented the adaptation?). **(If not clear on the role of the person named- see table below- clarify their role in the project. Record response and circle initial thoughts about which category in WHO domain in Table below best fits).**

1. WHEN during the TNP program was this adaptation first made?

PROBE: If early and if not clear, probe if was before or after began implementing. **(Record response and circle initial thoughts about which category in WHEN domain in Table below response best fits).**

1. HOW or on what BASIS was this change made?

PROBE: based on challenges implementing, on time concerns, on results or data you collected, on external or administrative concerns, feedback from patients or staff, or what basis? **(Record response and circle initial thoughts about which category in HOW domain in Table below best fits).**

1. WHY Part 1: WHY was this adaptation made?

PROBE: For example, to get more people to participate, to make the program attractive to more settings, to increase its effectiveness, to make it easier to deliver, to make it easier to maintain or reduce costs, etc.? **(Record response and circle initial thoughts about which category in WHY domain in Table below response best fits).**

WHY Part 2: 6a. Was this adaptation a result of EXTERNAL factors or INTERNAL issues?

PROBE: EXTERNAL factors (for example change in organizational policies, reimbursement changes) or INTERNAL issues (such as workflow, changes in staff or similar issues)? **(Record internal or external and note specifics)**

1. What was the short term IMPACT of this adaptation?

PROBE: Did it have highly visible results? For example did it result in more or less participation by patients, get more or fewer settings or staff involved, improve or decrease consistency of delivery, improve or reduce outcomes, reduce or increase time or costs? We understand that you may not have concrete outcomes results at this time – please tell us your best perception of the impact of this adaptation thus far **(Record response and circle initial thoughts about which category in IMPACT domain in Table below best fits).**

1. OTHER? Probe any other aspects of adaptations that emerge from Interviewee comments**. (Record those at bottom of table below)**

**Repeat steps and questions for Adaptation # 2 and possibly #3 if time permits. First remind interviewee of how they described this second (third) adaptation in their own words.**

Table 1: Conceptual Basis and Response Options for Coding for Questions

| Domain | Description | Source | Interview Questions & Response Options |
| --- | --- | --- | --- |
| Who | Person(s) who initiated the adaptation | Stirman | Who was primarily responsible for initiating this modification?   - Entire or most of team - Practitioner - Administrator - Researcher - Developer - Stakeholder - Coalition - Other |
| What  Part 1  What  Part 2 | Content of the intervention | Stirman | Which of the following elements was primarily changed as part of this adaptation?   - The setting - The format - Personnel involved - The target population - How the intervention is presented - Other |
|  |  |  | Which of the following was the primary type of change involved?   - Tailoring to individuals - Adding a component - Removing a component - Condensing a component - Extending a component - Substituting for a component - Changing the order of components - Integrating with other programs we are doing - Repeating a component - Loosening the structure or protocol - Otherwise changing the intervention |
| When | When during the project the adaptation was made | Study Team | At which of the following points in the project was this change FIRST made?   - During planning stages before began intervention - Early during first few weeks of intervention - During the middle stages - In the later stages - At or close to the end of project |
| How | How the adaptation was made and on what basis | Study Team | What was the primary basis on which this change was made?   - Based on our vision or values - Based on a framework (for example PCMH) - Based on our knowledge or experience of working with patients - Based on QI data, summary information or results - Based on pragmatic/practical considerations (for example “this is the only way it would work”) - Based on financial incentives/payment - Based on feedback or suggestions (Practice Facilitator/coach or other) - Other |
| Why | Reasoning behind the adaptation | RE-AIM | Which of the following was the primary reason behind this change?   - To increase he number or type of patients contacted (reach) - To enhance the impact or success of the intervention for all or important subgroups (effectiveness) - To make it possible to involve more teams, team members or staff (adoption) - To make the intervention delivered more consistently; to better fit our practice, patient flow or EHR; for practical reasons (implementation) - To institutionalize or sustain the intervention (maintenance) - To respond to external pressures or policy - To save money or other resources (implementation) - Other |
| Impact | Results of the adaptation, positive or negative | RE-AIM | Which of the following results or impact was the primary result of this adaptation?   - No major changes   Increased or decreased: (be sure to record which)  Increase_____ Decrease______   - Number or type of patients engaged (reach) - Quality of care or other outcomes (effectiveness) - Participation by teams or staff (adoption) - Consistent delivery of quality care or costs (implementation) - Maintenance or sustainability of the intervention in the practice (maintenance) - Maintenance or sustainability of the patient within the intervention (maintenance) - Reimbursement or financial implications for the practice - Efficiency (getting more done faster or with less resources) |

Other Comments, additional features of the adaption not covered above:

**Additional Feedback Questions for the Transitions Nurses Only**

Thank you so much for that information. Next, we would like to ask a few more questions to reflect on the TNP implementation process as you were rolling out the program at your site.

*Pre-Implementation Data Collection:*

1. What did you think about the site visit last winter? (probe on specific activities, if appropriate: observations, process mapping, brain-writing, team meeting)
   1. What do you think worked well?
   2. What did not?
   3. What could we do better?
2. Do you remember receiving written feedback after the visit? *(If no, describe the one-pager provided at the end of the site visit?*
   1. How, did this feedback inform your approach to getting the TNP started at your facility?
   2. How did this feedback help you understand how the TNP could fit into your local facility?
   3. Was there any information you wish you had had prior to getting TNP started at your facility?

*Community of Practice:*

1. What did you think about the two-day training in Denver?
   1. What did you think of the CAPE training?
   2. What did you think of the group sessions?
   3. What did you think of the binders that were provided as part of the training?
      1. Discharge process map?
   4. How did it meet your expectations and/or needs?
   5. How did it prepare you for your role as a Transitions Nurse?
   6. What could we do better?
2. Since starting the TNP, have you used any of the TNP implementation materials (e.g. pamphlets, educational PowerPoints, promotional posters, video, pocket card, etc.)?
   1. Grounded prompts
   2. Do you have any suggestions for how they could be improved?
3. Can you tell me about the weekly calls?
   1. How have they met your expectations and/or needs?
   2. Educational guidance?
   3. Individual guidance and support?

*Audit and Feedback*

1. Can you tell me your thoughts about the dashboard?
   1. Have you made any changes to the TNP as a result of the information provided from the dashboard?
   2. Is there additional information about the program that you would like to receive on regular basis?
2. Which aspects of the TNP implementation process have you found most important to help you prepare for your role as a TN?
   1. Which aspects helped you prepare for the TNP roll-out at your site?
3. Is there anything else you would like to share with us?

**Additional Feedback Questions for the Champions Only**

Thank you so much for that information. Next, we would like to ask several questions to reflect on the guidance and training you received as you were rolling out the program at your site.

*Pre-Implementation Data Collection:*

1. What did you think about the site visit last winter? (probe on specific activities, if appropriate: observations, process mapping, brain-writing, team meeting)
   1. What do you think worked well?
   2. What did not?
   3. What could we do better?
2. Do you remember receiving written feedback after the visit? *(If no, describe the one-pager provided at the end of the site visit?*
   1. How did this feedback inform how you approached getting the TNP started at your facility?
   2. How did this feedback help you understand how the TNP could fit into your local facility?
   3. Was there any additional information you wish you had received prior to getting TNP started at your facility?

*Community of Practice:*

1. What did you think about the two-day training in Denver?
   1. What did you think of the CAPE training?
   2. What did you think of the group sessions?
   3. What did you think of the materials that were provided as part of the training?
   4. How did it meet your expectations and/or needs?
   5. How did it prepare you to initiate the TNP at your site?
   6. What could we do better?
2. Can you tell me about the monthly Hospital Champion calls?
3. How have they met your expectations and/or needs?

*Audit and Feedback*

1. What about the dashboard?
   1. Have you made any changes to the TNP as a result of the information provided from the dashboard?
2. Is there additional information about the program that you would like to receive on regular basis?
3. Which aspects of the TNP implementation process have you found most important to help you prepare for your role as a clinical champion?
   1. Which aspects helped you prepare for the TNP roll-out at your site?
4. Is there anything else you would like to share with us?

**Additional Feedback Questions for the PACT Team Members Only**

**Note to the interviewer:**

Use the interview introduction and questions above at your discretion during the course of the interview. The PACT team members will have a limited view of the TNP project and adaptations but would still be able to provide valuable feedback about the TNP impact and inform future adaptations. Also, keep in mind the grounded probes to inquire about specific examples.

1. Please tell me about your experience with the TNP project?
   1. Please tell me your opinion about the TNP project
   2. PROBE: How has it impacted your role and responsibilities?
   3. PROBE: Have there been any changes made to your daily processes as a result of the TNP?
   4. PROBE: how has it impacted the follow up care coordination?
2. Please tell me about your interactions with the TN nurse, <name>?
   1. How working with the TN impacted transitioning Veterans after VAMC hospitalization?
   2. How often do you interact with the TN?
   3. How else can the TN be helpful to you?
   4. PROBE: What does the TN need to do differently, if anything?
3. Is there anything else you would like us to know about the TNP or the Transitions Nurse?
