# Supplementary material for: Understanding adaptations in the Veteran Health Administration’s Transitions Nurse Program: refining methodology and pragmatic implications for scale-up
Source: Implement Sci. 2021 Jul 13;16:71. doi: 10.1186/s13012-021-01126-y (PMC8276503; doi:10.1186/s13012-021-01126-y)
Supplement: Supplementary file 3 — Additional file 3. Appendix 3. Overall adaptations and unique adaptations per site and per data source. [file 13012_2021_1126_MOESM3_ESM.docx]

Appendix 3: Overall Adaptations and Unique Adaptations

|  | # interviews and data entries | | | # adaptations entries | | | | Total | # unique adaptations | | | | Total |
| --- | --- | --- | --- | --- | --- | --- | --- | --- | --- | --- | --- | --- | --- |
| Source | ML | E | TD | ML | E | MC | TD |  | ML | E | MC | TD |  |
| Site 1 | 1 | 2 | 4 | 2 | 2 | 1 | 4 | 9 | 2 | 1 | 1 | 4 | 8 |
| Site 2 | 2 | 2 | 0 | 2 | 4 | 0 | 0 | 6 | 2 | 4 | 0 | 0 | 6 |
| Site 3 | 1 | 2 | 4 | 2 | 5 | 0 | 4 | 11 | 1 | 3 | 0 | 4 | 8 |
| Site 4 | 2 | 2 | 2 | 3 | 5 | 0 | 2 | 10 | 3 | 4 | 0 | 2 | 9 |
| Site 5 | 2 | 2 | 5 | 4 | 4 | 0 | 5 | 13 | 2 | 3 | 0 | 5 | 10 |
| **Total** | 8 | 10 | 15 | 13 | 20 | 1 | 15 | 49 | 10 | 15 | 1 | 15 | 41 |
| ML = mid-line interview, E = exit interview, TD = Transitions Nurse database, MC = member check | | | | | | | | | | | | | |
